# Association between the quantitative characteristics of dual-energy spectral CT and cytoreduction surgery outcome in patients with advanced epithelial ovarian cancers: A prospective observational study

**DOI:** 10.1097/MD.0000000000037437

**Published:** 2024-03-08

**Authors:** Xiaojuan Xu, Yan Chen, Xinxin Zhang, Yilin Wang

**Affiliations:** a Department of Diagnostic Imaging, National Cancer Center/National Clinical Research Center for Cancer/Cancer Hospital, Chinese Academy of Medical Sciences and Peking Union Medical College, Beijing, China.

**Keywords:** advanced epithelial ovarian cancer, cytoreduction surgery, dual-energy spectral computed tomography

## Abstract

This study aimed to explore the association between the quantitative characteristics of dual-energy spectral CT and cytoreduction surgery outcome in patients with advanced epithelial ovarian carcinoma (EOC). In this prospective observational study, patients with advanced EOC (federation of gynecology and obstetrics stage III–IV) treated in the Department of Gynecological Oncology at our Hospital between June 2021 and March 2022 were enrolled. All participants underwent dual-energy spectral computed tomography (DECT) scanning 2 weeks before cytoreductive surgery. The quantitative data included peritoneal cancer index (PCI) determined by DECT, CT value at 70 keV, normalized iodine concentration, normalized water concentration, effective atomic number (effective-Z), and slopes of the spectral attenuation curves (slope λ Hounsfield unit). Fifty-five participants were included. The patients were 57.2 ± 9.8 years of age, and 72.7% were menopausal. The maximal diameter of tumors was 8.6 (range, 2.9–19.7) cm, and 76.4% were high-grade serous carcinomas. Optimal cytoreduction was achieved in 43 patients (78.2%). Compared with the optimal cytoreductive group, the suboptimal cytoreductive group showed a higher PCI (median, 21 vs 6, *P* < .001), higher 70 keV CT value (69.5 ± 16.6 vs 57.1 ± 13.0, *P* = .008), and higher slope λ Hounsfield unit (1.89 ± 0.66 vs 1.39 ± 0.60, *P* = .015). The multivariable analysis showed that the PCI (OR = 1.74, 95%CI: 1.24–2.44, *P* = .001) and 70 keV CT value (OR = 1.07, 95%CI: 1.01–1.13, *P* = .023) were independently associated with a suboptimal cytoreductive surgery. The area under the receiver operating characteristics curve of PCI and 70 keV CT value was 0.903 (95%CI: 0.805–1.000, *P* = .000) and 0.740 (95%CI: 0.581–0.899, *P* = .012), respectively. High PCI and 70 keV CT value are independently associated with suboptimal cytoreductive surgery in patients with advanced EOC. The PCI determined by DECT might be a better predictor for suboptimal cytoreduction.

## 1. Introduction

Epithelial ovarian cancer (EOC) is the fifth leading cause of cancer deaths among women of all ages and accounts for more deaths than any other gynecological malignancies,^[1,2]^ with a 5-year survival rate of around 40%.^[3]^ A majority of patients (60–70%) present at an advanced stage, that is, the International Federation of Gynecology and Obstetrics (FIGO) stage III or IV.^[4]^ Primary cytoreduction surgery followed by platinum-based adjuvant chemotherapy are the standard treatments for these advanced cases.^[4,5]^ However, for the majority of women present in an advanced stage, management remains one of the greatest challenges because they eventually develop chemotherapy resistance and ultimately succumb to the disease.^[4,5]^

There is a large body of evidence demonstrating that the volume of residual disease after the debulking procedure remains the single best predictive factor in survival,^[6,7]^ with much emphasis placed on achieving optimal cytoreduction (no gross visible residual disease or residual disease < 1 cm at the end of debulking surgery).^[8–10]^ Suboptimal surgery has a negative effect on survival, so treatment strategies to avoid unnecessary surgery should be considered.^[11,12]^ If suboptimal debulking is expected before surgery, neoadjuvant chemotherapy followed by interval debulking surgery could be an alternative.^[13]^ Therefore, the appropriate selection of patients for primary debulking represents a critical decision-making point in EOC management.

Clinical characteristics, tumor markers, hematologic indicators, radiologic images, and diagnostic laparoscopy have been applied to predict optimal debulking surgery in various studies.^[14–20]^ The establishment of models by combining imaging and diagnostic laparoscopy, represented by the peritoneal cancer index (PCI), which can reflect the distribution and degree of peritoneal metastasis of EOC semiquantitatively, is currently a widely recognized method for predicting optimal debulking.^[21]^ Nevertheless, these indicators are mainly semiquantitative, and no quantitative imaging biomarkers have been studied.

Dual-energy spectral computed tomography (DECT) is a new technology that simultaneously acquires images at 2 different photon spectra during a single CT acquisition. Compared to conventional single-energy CT, a DECT scan uses a single tube with fast and dynamic kVp switching between 80 and 140 kVp X-rays during a single rotation and generates 101 monochromatic CT images in the range of 40–140 keV and iodine/water-based density and effective atomic number images.^[22,23]^ Therefore, DECT can provide multiple quantitative measurements, including the monochromatic CT number, the slope of the spectral Hounsfield unit (HU) curve (slope λ HU) based on monochromatic images, the iodine concentration based on iodine-based density images, the water concentration based on water-based density images, and the effective atomic number (effective Z) based on effective atomic number images.

DECT has potential applications in various oncology areas, including differential diagnosis, prediction of lymph node metastasis, and prognosis evaluation.^[24–27]^ Concerning EOC, DECT has been used for distinguishing histological subtypes with higher accuracy than conventional CT.^[28–30]^ To the best of our knowledge, research focused on DECT characteristics with the debulking outcome is scarce. Therefore, this study aimed to explore the association between the quantitative characteristics of DECT and cytoreduction surgery outcome in patients with advanced EOC. The results could help a better selection of the patients for surgery.

## 2. Materials and methods

### 2.1. Study design and participants

In this prospective observational study, patients with advanced EOC (FIGO stage III-IV) treated in the Department of Gynecological Oncology, our Hospital between June 2021 and March 2022 were enrolled. This study was approved by the Ethics Committee of our Hospital. All participants signed the informed consent form before inclusion.

The inclusion criteria were (1) pathologically diagnosed with EOC, (2) classified as FIGO stage ≥ III by clinical examinations, tumor biomarker tests, and imaging examinations,^[31]^ (3) underwent primary cytoreduction surgery, without hyperthermic intraoperative chemotherapy, and (4) underwent DECT scanning within 2 weeks before the operation. The exclusion criteria were (1) with contraindications to contrast agents of CT or could not undergo enhanced CT scanning, or (2) the quality of the CT images was poor, or the primary lesions were not assessable. No patients underwent DECT outside our study center previously.

### 2.2. CT examination

All participants underwent DECT scanning 2 weeks before debulking surgery. The patients underwent intravenous contrast-enhanced DECT examination using a 256-detector row Revolution CT machine (GE Healthcare Milwaukee, WI). The participants took moderate contrast medium orally to make the intestine full about 4 to 6 hours before the examination. The gemstone spectral imaging (GSI) scan parameters included helical scan, dual kVp (80 and 140 kVp), momentary switched (0.5 ms), adaptive current, a detector coverage of 40 mm, a scan range from the diaphragm level to the inferior margin of the pubic symphysis, a section thickness of 5 mm, a reconstruction interval of 5 mm, a pitch of 1.375, and a gantry rotation speed of 0.5 s. The nonionic contrast media (Ultravist 300; Bayer Schering Pharma, Berlin, Germany) at 1.5 mL/kg was injected with a power injector at 2.5 mL/s through the median cubital vein. It was followed by a 20-mL saline flushing at 2.5 mL/s. The venous phases were obtained at a 65-s delay after contrast medium injection.

### 2.3. Image analysis

The monochromatic images with a slice thickness of 2.5 mm and spacing of 2.5 mm in the venous phases were transferred to an AW4.4 workstation (Advantage Workstation 4.6, GE Healthcare, Milwaukee, WI) for analysis using a special GSI viewer. The regions of interest (ROIs) were selected in the solid and enhancing component, avoiding hemorrhagic or cystic areas. Two doctors specializing in gynecological oncology with 10 and 5 years of gynecology tumor imaging experience, respectively, evaluated the images. In cases of discordant interpretations, decisions were reached by consensus. The radiological PCI (a clinical integration of both peritoneal implant size and distribution of nodules on the peritoneal surface, as described by Sugarbaker^[32]^) determined by DECT was assessed. Each lesion was measured 3 times, and the average value was used for analysis. The effective atomic number (effective-Z) of the lesion was recorded from the effective atomic number image. Normalized iodine concentration and normalized water concentration were determined from water and iodine-based MD images, respectively. The λHU slope was calculated using λHU slope = (CT 40 keV–CT 100 keV)/ (100–40). Meanwhile, the enhanced CT value in monochromatic images at 70 keV (CT value at 70 keV) was selected since conventional 120 kVp polychromatic images have similar mean energy values as 70 keV GSI images. Therefore, the quantitative data included CT values at 70 keV, normalized iodine concentration, normalized water concentration, effective-Z, and slope λ HU.

The maximum diameter of the tumor, configuration, surface, enhancement pattern, ascites, lymphadenopathy, and peritoneal implants were assessed from the DECT. The tumors were divided into solid (solid components > 70%), cystic (cystic components > 70%), and solid-cystic type (cystic components between 30% and 70%). The tumor surface was classified as smooth and irregular. The enhancement pattern was homogenous or heterogeneous. In the presence of peritoneal implants, the predominant morphologic pattern of peritoneal disease was defined as nodular (implants with predominantly well-defined or rounded or “pushing” borders) or infiltrative (implants with poorly defined or infiltrative borders). Lymphadenopathy was determined in the presence of (1) supradiaphragmatic lymph nodes > 0.5 cm, (2) portocaval lymph nodes > 1.5 cm, (3) porta hepatis (periportal) and suprarenal para-aortic lymph nodes > 1.0 cm, and (4) regardless of size if it had spiculated borders, heterogeneous attenuation, or if nodal clustering was present.

### 2.4. Cytoreductive surgery

All participants included in this study underwent primary cytoreductive surgery performed by 2 gynecologic oncologists (with 6 and 15 years of experience, respectively). A longitudinal incision was made at the lower abdomen, through which the tumors in pelvic and abdominal cavities were explored extensively. Panhysterectomy, bilateral salpingo-oophorectomy, and omentum majus resection were performed. All resectable, enlarged and suspect lymph nodes were resected. For patients with a visible suspected appendix surface, a tumor affecting the mesentery, or mucinous ovarian cancer, an appendectomy was performed. In order to achieve satisfactory cytoreductive effects, bowel resection, splenectomy, cholecystectomy, hepatectomy, and cystectomy, were performed according to the locations of metastatic lesions.

### 2.5. Surgical outcome

The primary surgical outcome of this study was the cytoreductive effect, which was assessed by the referring surgeon when the surgery was completed according to the presence of residual lesions and the sizes of the residual lesions. Optimal cytoreduction was defined as no residual tumor or a residual tumor < 1 cm; a > 1cm residual tumor was considered suboptimal cytoreduction.

### 2.6. Statistical analysis

SPSS 21.0 (IBM, Armonk, NY) was used for statistical analysis. Continuous data with a normal distribution were described as means ± standard deviation and analyzed using the independent *t*-test. Continuous data not in a normal distribution were described as medians (ranges) and analyzed using the Mann–Whitney U-test. Categorical data were described as n (%) and analyzed using the chi-square or Fisher exact test. For the multivariable analyses, the cytoreductive effect was used as the dependent variable, and factors with statistical significance in the univariable analyses were used as the independent variables. A receiver operating characteristics curve was used to examine the predictive value of the identified factors for cytoreduction effects. Two-sided *P*-values < 0.05 were considered statistically significant.

## 3. Results

**Figure 1.**
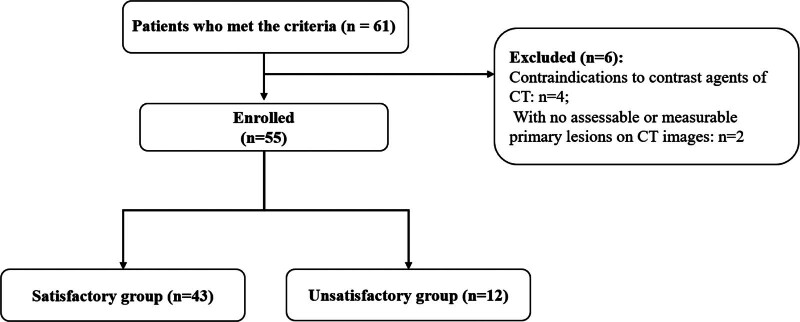
Participant flowchart.

Figure 1 presents the participant flowchart. Fifty-five participants were included. The participants were 57.2 ± 9.8 years of age, and 72.7% were menopausal. The maximal diameter of tumors was 8.6 (range, 2.9–19.7) cm, and 76.4% were high-grade serous carcinomas (Table 1). There were no differences between the optimal and suboptimal cytoreduction groups regarding age, menopause, maximal tumor size, and pathological subtype (all *P* > .05), but the CA125 levels were higher in the suboptimal cytoreductive group (*P* = .050).

**Table 1:** Characteristics of the participants.

Characteristics	All (n = 55)	Optimal cytoreduction (n = 43)	Suboptimal cytoreduction (n = 12)	*P*
Age (years)	57.2 ± 9.8	56.5 ± 10.3	59.5 ± 8.0	.354
Postmenopausal women	40 (72.7%)	29 (67.4%)	11 (91.6%)	.147
CA125	689.00 (11.83–7434.00)	629.50 (11.83–4674)	1453.50 (61.09–5354.00)	.050
Maximal diameter of tumor, cm	8.58 (2.90–19.68)	8.58 (3.19–19.68)	8.51 (2.90–10.90)	.234
Pathological subtype				.340
High-grade serous carcinoma	42 (76.4%)	31 (72.1%)	11 (91.7%)	
Low-grade serous carcinoma	1 (1.8%)	1 (2.3%)	0	
Endometrioid carcinoma	4 (7.3%)	4 (9.3%)	0	
Clear cell carcinoma	2 (3.6%)	1 (2.3%)	1 (8.3%)	
Mucinous carcinoma	6 (10.9%)	6 (14.0%)	0	

The data are presented as mean ± standard deviation, median (range), or n (%).

Compared with the optimal cytoreductive group, the suboptimal cytoreductive group showed a higher PCI (median, 21 vs 6, *P* < .001), higher 70keV CT value (69.5 ± 16.6 vs 57.1 ± 13.0, *P* = .008), and higher slope λ HU (1.89 ± 0.66 vs 1.39 ± 0.60, *P* = .015) (Table 2). There were no significant differences in morphological indexes between the 2 groups.

**Table 2:** Comparison of imaging characteristics between the 2 groups.

Characteristics	All (n = 55)	Optimal cytoreduction (n = 43)	Suboptimal cytoreduction (n = 12)	*P*
PCI	0–23 (8)	6 (0–19)	21 (8–23)	<.001
Tumor surface				.261
Smooth	25 (45.5%)	20 (46.5%)	5 (41.7%)	
Irregular	30 (54.6%)	23 (53.5%)	7 (58.3%)	
Tumor configuration				.708
Solid	24 (43.6%)	18 (41.9%)	6 (50.0%)	
Solid-cystic	26 (47.3%)	20 (46.5%)	6 (50.0%)	
Cystic	5 (9.1%)	5 (11.6%)	0	
Enhancement type				>.999
Homogenous	15 (27.3%)	12 (27.9%)	3 (25.0%)	
Heterogeneous	40 (72.7%)	31 (72.1%)	9 (75.0%)	
Peritoneal Implants	53 (96.4%)	41 (95.4%)	12 (100%)	.600
Type of peritoneal implants (n = 53)				.067
Nodular	27 (50.9%)	21 (51.2%)	6 (50.0%)	
Infiltrative	26 (49.1%)	20 (48.8%)	6 (50.0%)	
Ascites	39 (70.9%)	28 (65.1%)	11 (91.7%)	.147
Lymphadenopathy	13 (23.6%)	8 (18.6%)	5 (41.7%)	.129
70 keV CT value	59.84 ± 14.61	57.13 ± 12.95	69.54 ± 16.61	.008
NIC	0.26 ± 0.10	0.24 ± 0.09	0.27 ± 0.03	.356
NWC	1.00 (0.98–1.03)	1.00 (0.98–1.03)	1.00 (0.99–1.02)	.137
Effective-Z	8.36 ± 0.32	8.44 ± 0.25	8.53 ± 0.30	.065
Slope λ HU	1.50 ± 0.64	1.39 ± 0.60	1.89 ± 0.66	.015

The data are presented as mean ± standard deviation, median (range), or n (%).

The results of the multivariable analysis showed that the PCI (OR = 1.74, 95%CI: 1.24–2.44, *P* = .001) and 70keV CT value (OR = 1.07, 95%CI: 1.01–1.13, *P* = .023) were independently associated with a suboptimal cytoreductive surgery (Table 3).

**Table 3:** Multivariable analysis for suboptimal cytoreductive surgery.

Characteristics	OR	95%CI	*P*
PCI	1.739	1.237–2.444	.001
70 keV CT value	1.069	1.009–1.132	.023
Slope of energy spectral curve	1.131	0.069–18.536	.931

CI = confidence interval, CT = computed tomography, OR = odds ratio, PCI = peritoneal cancer index.

**Figure 2.**
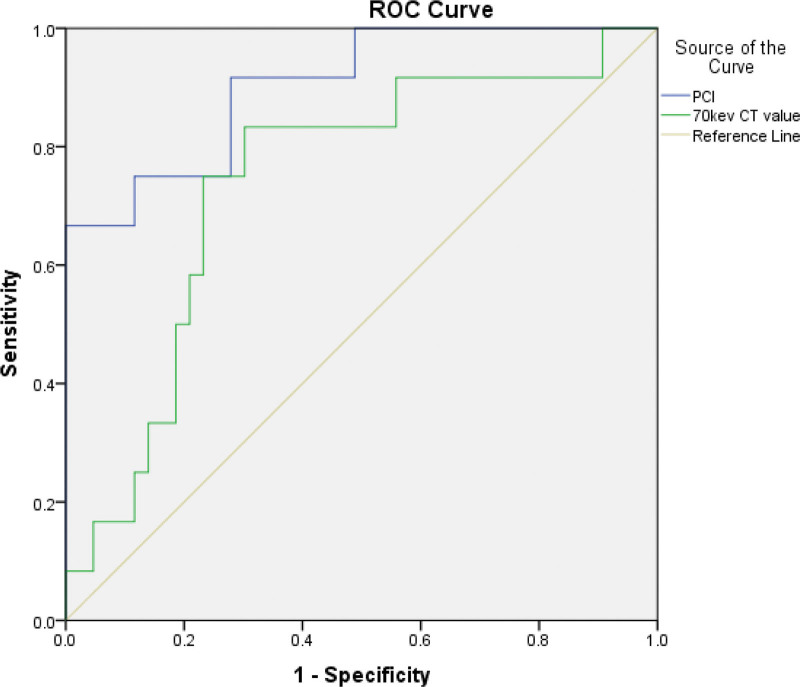
Receiver operating characteristics (ROC) curve of peritoneal cancer index (PCI) and 70 keV computed tomography (CT) value on cytoreduction effects.

The area under the receiver operating characteristics curve of PCI and 70 keV CT value was 0.903 (95%CI: 0.805–1.000, *P* = .000) and 0.740 (95%CI: 0.581–0.899, *P* = .012), respectively (Fig. 2).

## 4. Discussion

The results showed that high PCI determined by DECT and high 70-keV CT value are independently associated with suboptimal cytoreductive surgery in patients with advanced EOC. The PCI might be a better predictor for suboptimal cytoreduction.

Previous studies examined the clinical characteristics, tumor markers, hematologic indicators, radiologic images, and diagnostic laparoscopy to try to predict optimal debulking surgery.^[14–20]^ Suidan et al^[14]^ built a prognostic model using 3 clinical characteristics (age, CA125, and American Society of Anesthesiologists physical status 3–4) and 6 radiological features (suprarenal retroperitoneal lymph node > 1 cm, diffuse small bowels adhesion or thickening, and lesions > 1 cm in the small bowel mesentery, the root of the superior mesenteric artery, perisplenic area, and lesser sac) that achieved an AUC of 0.758. The biochemical markers CA125 and HE4 achieved AUCs around 0.70 for the outcome of cytoreductive surgery.^[15]^ A surgical score system by Kasper et al^[16]^ and based on American Society of Anesthesiologists 3–4, tumor presence in multiple numbers and multiple compartments, and the number of involved organs achieved an AUC of 0.91 despite the fact that this score is based only on macroscopic lesions.

In the present study, the PCI and the 70-keV CT value were independently associated with the outcome of cytoreductive surgery, and their AUCs were 0.90 and 0.74, respectively. Interestingly, Engbersen et al^[17]^ reported that the MRI-PCI had AUCs of 0.92–0.98 (depending upon the reader) for predicting complete cytoreduction, supporting the present study even though the present study was performed using DECT and not MRI. Still, Abdalla Ahmed et al^[20]^ showed that CT correlated well with the laparoscopic findings, while Goswami et al^[33]^ showed that the CT-PCI correlated well with the surgical PCI. Garcia Prado et al^[18]^ also reported that the MRI-PCI could predict complete cytoreduction, albeit with slightly lower accuracy. Still, the PCI could be combined with other parameters to improve accuracy. Indeed, Muallem et al^[19]^ reported that the combination of CA125, PCI, and intraoperative mapping of ovarian cancer score could predict complete cytoreduction in 90% of the patients.

The present study used DECT, which has potential applications in oncology.^[24–27]^ The use of DECT could have value for EOC,^[34,35]^ but studies are still necessary.^[29,34]^ The prospects of DECT in oncology are good as it showed value for differential diagnosis, prediction of lymph node metastasis, and prognosis evaluation of gastric,^[24,27]^ adrenal,^[25]^ and thyroid^[26]^ cancers. In EOC, DECT has a higher value than conventional CT in distinguishing the histological subtypes.^[28–30]^ In the present study, only the 70-keV CT value was associated with the outcome of cytoreductive surgery, which was not reported before. The difference between the 70-keV CT value and conventional single-energy CT scan might reflect some internal differences between tumor cells and normal tissues, but there are few relevant studies at present. A study in gastric cancer suggests DECT parameters correlate with Ki-67 expression, a marker of cell proliferation.^[24]^ There are also few studies on DECT in ovarian cancer, and most of the available studies are on the classification of benign and malignant ovarian tumors^[29,30,34]^ or on monitoring the effect of systemic treatments on abdominal tumors,^[36]^ but there are no studies on cytoreductive surgery. Therefore, the present study adds important information and an additional potential use for DECT in EOC. Additional studies might reveal other uses for DECT in EOC.

This study has limitations. Only 1 center participated in the study, and the sample size was relatively small. Second, further classification of the pathological subtypes based on molecular biology was not performed. There were multiple pathological subtypes of EOCs included in this study. Although high-grade serous carcinomas represented most cases, several other pathological subtypes, such as low-grade serous carcinoma, endometrioid carcinoma, and mucinous carcinoma, were also included. These subtypes might have different imaging characteristics, malignancy degrees, cytoreductive effects and outcomes, and the association of imaging characteristics. Future studies should focus on a single subtype. Third, the ROIs were only selected on 1 layer, and the information could be more abundant, and data could be more accurate if ROIs were selected on 3D layers. Fourth, only images of the venous phase were investigated in this study for pelvic and abdominal cavities. Although the blood supply features of ovarian cancer are different from tumors of the liver, pancreas, and kidney, dual-phase scanning could not provide additional information for the differential diagnosis of ovarian cancer.^[37]^ In addition, it is recommended that the X-ray dose on the patients should be minimized.^[37]^ Therefore, only venous phase scanning was performed in this study, but it is unclear whether the energy spectral parameters of arterial phase scanning could influence the findings, which need to be investigated in future studies.

In conclusion, this study showed that in patients with EOC and undergoing DECT before cytoreductive surgery, high PCI and high 70keV CT value are independently associated with suboptimal cytoreductive surgery. The PCI determined by DECT might be a better predictor for suboptimal cytoreduction. These results must be validated in multicenter studies with a large sample size. Other imaging modalities and parameters also need to be explored.

## Author contributions

**Conceptualization:** Xiaojuan Xu, Yan Chen.

**Formal analysis:** Xiaojuan Xu, Yan Chen, Xinxin Zhang, Yilin Wang.

**Methodology:** Xinxin Zhang, Yilin Wang.

**Writing – original draft:** Xiaojuan Xu.
